# Influence of Physical Activity and Ambient Temperature on Hydration: The European Hydration Research Study (EHRS)

**DOI:** 10.3390/nu8050252

**Published:** 2016-04-27

**Authors:** Ricardo Mora-Rodriguez, Juan F. Ortega, Valentin E. Fernandez-Elias, Maria Kapsokefalou, Olga Malisova, Adelais Athanasatou, Marlien Husemann, Kirsten Domnik, Hans Braun

**Affiliations:** 1 Exercise Physiology Lab at Toledo, University of Castilla-La Mancha, Toledo 45071, Spain; Juanfernando.ortega@uclm.es (J.F.O.); valentin.fernandez@uclm.es (V.E.F.-E.); 2 Department of Food Science and Human Nutrition, Agricultural University of Athens, Athens 11855, Greece; kapsok@aua.gr (M.K.); olgamalisova@yahoo.gr (O.M.); dathanasatou@gmail.com (A.A.); 3 Institute of Biochemistry, German Sport University, Cologne 50993, Germany; m.husemann@biochem.dshs-koeln.de (M.H.); k.domnik@biochem.dshs-koeln.de (K.D.); H.Braun@dshs-koeln.de (H.B.)

**Keywords:** hydration status, physical activity, urine osmolality, 24-h urine volume

## Abstract

This study explored the effects of physical activity (PA) and ambient temperature on water turnover and hydration status. Five-hundred seventy three healthy men and women (aged 20–60 years) from Spain, Greece and Germany self-reported PA, registered all food and beverage intake, and collected 24-h urine during seven consecutive days. Fasting blood samples were collected at the onset and end of the study. Food moisture was assessed using nutritional software to account for all water intake which was subtracted from daily urine volume to allow calculation of non-renal water loss (*i.e.*, mostly sweating). Hydration status was assessed by urine and blood osmolality. A negative association was seen between ambient temperature and PA (*r* = −0.277; *p* < 0.001). Lower PA with high temperatures did not prevent increased non-renal water losses (*i.e.*, sweating) and elevated urine and blood osmolality (*r* = 0.218 to 0.163 all *p* < 0.001). When summer and winter data were combined PA was negatively associated with urine osmolality (*r* = −0.153; *p* = 0.001). Our data suggest that environmental heat acts to reduce voluntary PA but this is not sufficient to prevent moderate dehydration (increased osmolality). On the other hand, increased PA is associated with improved hydration status (*i.e.*, lower urine and blood osmolality).

## 1. Introduction

Water intake comes from drinking fluids (water and other beverages), moisture in food and water produced by the body during oxidation. In turn, body water losses occur via urine, feces, sweat and insensible loss through the skin and by evaporation from the respiratory tract. Hydration status is the result of the balance between water intake and body water loss. When body water losses are higher than fluid intake, hypohydration results. In this paper, we will refer to the acute process of loss of body water as dehydration [1] and to the maintained body fluid deficit as hypohydration. Hypohydration has been linked to negative long-term health outcomes. Inadequate hydration together with elevated ingestion of calcium and sodium are related to nephrolithiasis [2]. A low intake of plain water is also associated with a higher prevalence of chronic kidney disease [3]. Furthermore, low water intake is associated with increased risk of developing hyperglycemia [4] and may increase the risk of developing type II diabetes [5]. Prospective studies measuring the impact of increased water intake on the development of these diseases are still in a preliminary phase [6].

Adequate fluid intakes (AI) representing population median consumption in apparently healthy sedentary adults under temperate climate have been reported [7,8]. The Institute of Medicine guidelines for adequate intake of total water for USA and Canada is 3.7 and 2.7 L/day for men and women, respectively [8]. However, the European Food Safety Authority (EFSA; [7]) has lower total water intake recommendations of 2.5 and 2.0 L/day for adult men and women, respectively. The EFSA adequate intake calculations attempted to incorporate hydration status into the recommendation and account for the maintenance of daily urine osmolality below a certain threshold (<500 mOsmol·kg^−1^; [7]). Although general recommendations for adults exist, both organizations recognize that individual requirements could widely vary depending on personal characteristics (age, size, body composition, physical activity) and environment.

The effects of physical activity on fluid intake and loss in free-living adults has been previously investigated in a small sample of healthy young lean subjects in The Netherlands [8]. In that study a sample of 42 women and 10 men were tested for water intake (weighted record of foodstuff and beverage) and physical activity (difference between total and resting energy expenditure) in summer and winter [9]. The study revealed that in men, water loss was proportional to physical activity, but in women water loss was higher in summer but was unrelated to physical activity [9]. In another study, a comparison of water turnover in rural and urban women in Kenya revealed that BMI was the stronger predictor of water loss but the addition of physical activity (measured by accelerometry) explained an additional 12% of the variance [10]. Thus, the influence of physical activity level on water loss/intake is not readily evident from the available literature. Furthermore, no measure of hydration status (changes in blood or urine concentration) was reported in these studies. In addition, the interplay between climate and physical activity and its consequences for water loss and intake are largely unknown in the European population.

Some studies suggest that the fluctuations in climate, food and water availability linked to seasons may be an important risk factors for malnutrition and other disorders [11]. The identification of seasonal differences in fluid intake [12], and the effect of environmental temperature on fluid loss could be relevant for the implementation of season-specific strategies to improve the hydration habits of the population if deficiencies are detected. Body water needs are highly individualized and depend upon body size and composition, resting metabolic rate, physical activity, dietary osmotic load and climate among others [13]. The purpose of this study was to assess the influence of physical activity and environmental temperature (linked to season) on water intake, water losses and hydration status in a large sample of healthy men and women aged 20–60 years from one northern (Germany) and two southern (Spain and Greece) European countries. We used a series of objective (body weight, blood and urine chemistry) and subjective (questionnaires and diaries) measures to assess hydration status and water intake and output, respectively. A novel characteristic of our study is that we followed subjects during seven consecutive days in an attempt to increase accuracy and spread possible spurious reporting into a more extensive data collection set.

## 2. Experimental Section

### 2.1. Participants

Between winter 2013 and summer 2014, a sample of 573 men and women from Germany, Spain and Greece were studied during seven consecutive days of free-living. Subjects were healthy and not disabled according to a pre-participation medical questionnaire, not undergoing a diet, not taking medicines that could affect the outcome measures (diuretics, phenytoin, lithium, demeclocycline or amphotericin B) and women were non-pregnant or breastfeeding and were tested out of their proliferative menstrual phase. Subject recruitment was oriented to reach a quota of 25 subjects in each of the following age groups in each country; 20–30; 31–40; 41–50; 50–60 years old. This subject recruitment scheme (100 per country) was repeated in winter and summer with a goal of 200 subjects tested per country. All centers obtain ethical approval from their local Institutional Review Board and all subjects signed an informed consent form were the study was detailed (197/27-02-2012 for Agricultural University of Athens, Greece, 4/02/2013-18 for University of Castilla-La Mancha, Spain, 1/26-11-2012 for German Sport University, Germany).

### 2.2. Study Design

Upon recruitment, subjects were instructed on dietary and fluid intake data logging and 24-h urine collection procedures and were given the materials necessary for these tasks. On the morning of the first and the last day of testing (day 1 and day 8) subjects arrived at the laboratory after an overnight fast and were weighed with only their underwear using a sensitive scale (±0.05 kg) before a blood sample was collected. For the remaining of the week subjects collected all urine produced, logged every foodstuff and beverage ingested at the time when it occurred and filled in a questionnaire that summarized their physical activity at the end of every day.

### 2.3. Food and Water Intake Diary

Total water intake corresponded to the sum of beverages and water in food recorded from the dietary records of each day of the testing week. Water content in the food was analyzed with nutritional software specific for each population (*i.e.*, CESNID, Barcelona, Spain; ESHA Research, Wadsworth Publishing Co Inc, Salem, OR, USA for Greece; and EBIS pro German Food Database 3.1, University of Hohenheim, Stuttgart, Germany).

### 2.4. Physical Activity Diary

Physical activity was evaluated using the short version of the International Physical Activity Questionnaire (IPAQ) [14]. The questionnaire requires recording of the minutes per day of vigorous, moderate, walk or sitting time and the days per week of each activity. The questionnaire was filled out daily, but was reduced to four questions by suppressing the frequency questions (*i.e.*, days per week) since data was collected every day. IPAQ data was processed in a continuous mode [14,15] accounting for 3.3 metabolic equivalent of a task (MET) for walking, 4.0 METs for moderate activities and 8.0 METs for vigorous activities. The units were expressed as METs-min per week as recommended by the investigators who validated the questionnaire [14,16,17].

### 2.5. Urine and Blood Biomarkers

Subjects arrived at the laboratory in the morning of the first day of testing after an overnight fast. Subjects provided a sample of their first morning urine and were weighed. After 15 min in a seated or a reclined position a 5 mL blood sample was drawn from an antecubital vein. A 0.5 mL aliquot was immediately analyzed for hemoglobin (ABL-520, Radiometer, Bronshoj, Denmark) hematocrit in duplicate by microcentrifugation (Biocen, Alresa; Barcelona, Spain). The remaining blood was allowed to clot in serum tubes (Z Serum Sep Clot Activator, Vacuette, Kremsmunster, Austria) centrifuged at 2000 g for 10 min (MPW-350R, Medical Instruments, Warsaw, Poland). The so obtained serum portion was analyzed for osmolality by freeze point depression (Advanced Instruments, Norwood, MA, USA) and the remaining stored at −30 °C for further analyses. Serum sodium and potassium concentrations were analyzed using an ion selective analyzer (Easylyte Plus, Medica Corporation, Bedford, MA, USA) and glucose concentration by spectrophotometry using glucose oxidase to produce gluconate from glucose (Thermo Scientific, Waltham, MA, USA). Subjects were then dispatched and advised to proceed with their normal life routines during the following 7 days while recording all food and fluid ingested and collecting all urine produced. On the morning of day 8, subjects returned to the lab for body weight, blood sampling and morning urine void. During the seven days of urine collection, subjects weighed and recorded every void with the aid of a collection vessel and a portable scale (±1 g accuracy). Then, they saved in a plastic bag a representative aliquot (~3 mL) of every void, labeled with the date, time of day and subject initials. The bag with the aliquots were kept refrigerated by the subjects. Urine from each day was reconstituted in proportion to the volume urinated and analyzed for osmolality, sodium and potassium using the same instruments described for blood analyses. Only one laboratory analyzed serum osmolality within 1 hour of blood collection and before freezing the serum samples while blood storage was the norm in the other two laboratories. Since variability due to different analyses time was a concern [18,19] we calculated serum osmolality according to the following formulae:

(1) Calculated Osmolality = 2 × ([Na^+^] + [K^+^]) + ([Glucose])

where all concentrations are in mmol/L.

### 2.6. Hydration Status Calculations

We assumed that our subjects were in water balance and therefore total water intake matched total water losses. Any cumulative discrepancy would have resulted in a change in body mass that should have been apparent in a change in body mass measured on day 8. The difference between water intake from the dietary records and 24-h urine volume represented non-renal water losses (NRWL) composed of sweat, respiratory and fecal water. We calculated the 7 days average NRWL as follows [20]:

(2) NRWL (L/day) = average total water intake (L/day) − average 24-h urine volume (L/day)

The excretion of solutes by the kidneys per day is the product of the average urine osmolality (mOsm/kg) multiplied by urine volume in liters per day. Since our subjects weighed their urine voids for this calculation, we assumed that 1 kg urine corresponds to 1 L and that errors introduced by the variable specific gravity of urine will be small. Obligatory urine volume (OUV) is the water volume necessary to excrete all urine solutes. To calculate OUV, a threshold of maximum urine osmolality of 830 mOsm/kg is used for a 20 years old individual [21]. Since aging reduces the renal capacity to concentrate urine [21], 3.4 mOsm/kg are subtracted from that threshold value per year above 20 years. The calculation then is as follows [22]:

(3) OUV (L/day) = average 24-h urine solutes (mOsm/day)/[830 − 3.4 × (age − 20)]

Free-water reserve (FWR) is the difference between the actual 24-h urine volume and the calculated obligatory urine volume [23]:

(4) FWR (L/day) = 24-h urine volume (L/day) − obligatory urine volume (L/day)

Positive FWR values reflects euhydration and negative values hypohydration. In a population, euhydration is ensured if at least 97% of the subjects show positive values of free-water-reserve [22]. We also categorized subjects as hypohydrated if their urine osmolality exceeded the 500 mOsm/kg threshold based on EFSA recommendations [7].

### 2.7. Statistical Analysis

Normality of data distribution was evaluated for each variable using parametric Shapiro-Wilk test. Data collected during seven consecutive days (urine output, fluid intake, peak ambient temperature and physical activity) were averaged in each subject. Subjects missing some day value in one variable were not excluded from the analysis and the average of the week value was calculated from the remaining data. Subjects missing more than two values in a given variable were removed from the analysis in that variable. Data collected at the beginning and end of the experiment (morning of day 1 and day 8) were also averaged (body weight and blood chemistry) after checking for variability (Table 1). Discrete variables with two levels (gender, season, free water reserve) were analyzed using student’s *T*-test for unpaired samples. Discrete variables with more than two levels (country, age group) were analyzed using ANOVA. Upon a significant *F* value Tukey’s post-hoc was used to identify differences between groups. Cohen’s formula for effect size (ES) [24] was used, and the results were based on the following criteria; >0.70 large effect; 0.30–0.69 moderate effect; ≤0.30 small effect. Associations between continuous variables were tested using Pearson product-moment correlation coefficient (*r*). Partial correlation was used to adjust for age as a covariate. All tests were performed with SPSS software version 18 (IBM Software, Chicago, IL, USA). Data are presented as mean ± SD. All statistical test were two-tailed and statistical significance level was set at *p* < 0.05.

## 3. Results

Table 2 depicts the number of subjects for each discrete category in the main variables of interest in this study, *i.e.*, physical activity and maximal air temperature. An average of 536 of the 573 subjects that completed the study (93%) had data in these two variables. In addition, data were well balanced between the category levels such as gender, season, country, age group (see percentages in Table 2). We analyzed the variability, one week apart, for body weight and some blood parameters. After one week of data collection, blood hemoglobin (14.7 ± 1.7 *vs.* 14.7 ± 1.6 g/L) and glucose (4.8 ± 1.8 *vs.* 4.7 ± 0.6 mmol/L) remained unchanged (*P* > 0.05). Body weight (74.6 ± 15.4 *vs.* 74.4 ± 15.3 kg), blood sodium (143 ± 8 *vs.* 141 ± 6 mmol/L) and potassium (4.50 ± 0.52 *vs.* 4.46 ± 0.46 mmol/L) were slightly but significantly reduced after one week of testing (*p* < 0.05). However, the differences were very small judging by Cohen’s effect size calculation (≤0.30 small effect). Thus, we feel entitled to average data during the seven consecutive days of testing to improve the power analysis and data consistency.

Physical activity (PA) in METs-min per week was not affected by gender (*p* = 0.065; ES = 0.132; Table 1) or age group (*p* = 0.410). However PA was lower in the summer (*p* < 0.001; ES = 0.388) and higher in individuals better hydrated with urine osmolality below 500 mOsmol/kg (*p* = 0.008; ES = 0.249) and positive free water reserve (*p* = 0.019; ES = 0.259). There was also a marked country effect on physical activity with Germans reporting higher levels than Spaniards and Greeks (both *p* = 0.001; ES = 0.532 and 0.905, respectively). In turn, Spaniards reported higher physical activity than Greeks did (*p* = 0.001; ES = 0.605; Table 1). Physical activity data was positively associated with urine volume and water intake while negatively associated with urine, blood osmolality and maximal air temperature (Table 3 and Table 4).

As expected, the maximal seven day average air temperature was higher in the summer than in winter (*p* < 0.001; ES = 2.827; Table 1). Maximal temperatures were lower in Germany than in Spain and Greece (both *p* = 0.001; ES = 0.751 and 0.877, respectively) without differences between these two south European countries. Maximal air temperature was lower in the better-hydrated subjects, those with urine osmolality below 500 mOsmol·kg^−1^ (*p* < 0.001; ES = 0.337) and in positive free water reserve (*p* < 0.001; ES = 0.430; Table 1). Maximal average air temperature was positively correlated with non-renal fluid loss (*i.e.*, sweating) and urine osmolality (*r* = 0.147 and 0.218, respectively; *p* < 0.001) and negatively with urine volume (*r* = −0.222; *p* < 0.001; Table 3).

Average 24-h water intake was higher in men and in all subjects during the summer (Table 1). Furthermore, water intake was lower in Spain and Greece than in Germany but not different among age groups (Table 1). Interestingly, water intake was positively correlated with physical activity, urine volume, non-renal water losses (*i.e.*, NRWL) and negatively correlated with urine osmolality (*r* = −0.349; *p* < 0.001; Table 3). Likewise, with 24-h water intake, average 24-h urine volume was higher in the German subjects than in the Spaniards and Greeks. We observe that increases in age are associated to an increase in urine volume (from 40 to 60 years’ old) and a reduction in urine osmolality (Table 1). Urine volume was positively correlated with physical activity (*r* = 0.152; *p* < 0.001) and water intake (*r* = 0.623; *p* < 0.001) and negatively with maximal air temperature (*r* = −0.222; *p* < 0.001) NRWL and urine osmolality (*r* = −0.736; *p* < 0.001; Table 3).

NRWL were higher in males and in the summer (Table 1). NRWL were positively associated with maximal temperature (*r* = 0.147; *p* < 0.001), water intake (*r* = 0.716; *p* < 0.001), negatively with urine volume (*r* = −0.095; *p* < 0.024) and positively with urine osmolality (0.208; *p* < 0.001; Table 3). However, NRWL was not associated with physical activity or serum osmolality.

Average 24-h urine osmolality (an index of hydration, [25]) was higher in males, lower in the German sample and as mentioned above decreasing with the increases in age group (see last column of Table 1). Based on urine osmolality we calculated obligatory urine volume and the difference with the actual urine volume collected to result in the calculation of free water reserve (*i.e.*, FWR). FWR was negative in 29% of the sample (163 out of 558 subjects with this variable) which suggests hypohydration in an important portion of our sample. Subjects with negative FWR (*i.e.*, hypohydrated) ingested less fluid on a daily basis, had lower urine output, lived in higher environmental temperatures, had higher NRWL (*i.e.*, sweat) and higher urine osmolalities (Table 1).

## 4. Discussion

There are several studies in the literature using dietary and activity logs to determine water intake and physical activity levels in different populations [9,10,26,27,28]. The novelty of our data is that although we use these subjective measurements of physical activity and water intake we followed subjects during seven consecutive days in an attempt to increase accuracy and spread possible spurious reporting into a more extensive data collection set. Subjects were instructed to carry with them their meal and beverage log and to report their physical activity at the end of every day by completing four simple questions modified from the international physical activity questionnaire (*i.e.*, short version IPAQ, [14,17,29,30]). Expectedly, the differences between weekend and weekdays in physical activity, fluid and meal ingestion were normalized by the full week collecting period with subjects starting data collection at different days of the week. In addition, subjects collected 24-h urine output during those seven consecutive days and a fasting blood sample was drawn in the morning of day 1 and day 8. These biological samples provide us with objective data to determine body hydration based on urine and blood osmolality. Using urine output in conjunction with water intake diaries we calculated non-renal water losses (NRWL) assuming that subjects were in fluid balance during the week of testing. Based on average urine osmolality we also calculated obligatory urine volume and free water reserve (FWR; see methods) to enhance our ability to detect dehydration using different indexes.

### 4.1. Effects of Physical Activity on Water Intake/Fluid Loss and Hydration

We found that physical activity estimated by seven days average IPAQ, is negatively associated with elevations in dehydration indexes (urine and blood osmolality; Table 3 and Table 4). This suggests that high levels of physical activity does not increase the risk of hypohydration. Conversely, our less physically active individuals are more likely to be hypohydrated based on urine osmolality. Physical activity seems to increase water turnover since we also found a positive association between physical activity and water intake and urine volume (Table 3). The transitory dehydration that accompanies increases in physical activity triggers the release of hormones like arginine vasopressin, which in turn stimulates thirst to regain fluid balance. In our data, 24-h water intake was strongly correlated with non-renal water loss (*i.e.*, sweating; *r* = 0.716; *p* < 0.001) which suggest that increased levels of physical activity are met by increased water intake in a voluntary or thirst-induced response to restore the water deficit created by exercise. Seemingly, elevated levels of physical activity result in a higher water loss compensated with higher fluid intake, which prevents from dehydration and even seems to promote a better hydration status (lower urine and blood osmolalities). There may be non-physiological influences for the observed increased water consumption in people with higher levels of physical activity. Among those, consumer education throughout advertisement that permeates and convince exercise enthusiast to increase hydration beyond their thirst drive.

It is well known that repeated bouts of vigorous physical activity results in hemodilution [31] due to plasma volume expansion [32]. However, it is unclear if the moderately-intense physical activity of most of our subjects (grand mean of 2241 MET-min·week^−1^) could result in plasma volume expansion. Plasma volume expansion results if physical activity is followed by thermally induced profuse sweating (*i.e.*, sauna, [33]). Thus, it is possible that the combination of moderately-intense physical activity of our subjects and the exposure to high ambient temperatures in the summer could have resulted in some degree plasma volume expansion. We have recently found that exercise training expands not only blood plasma but also water within the exercised muscles [34]. Thus, the finding that people with increased physical activity show reduced urine osmolality could be explained by this exercise training adaptations that raise body water content. Suggesting plasma volume expansion, blood concentration of sodium, potassium, glucose and calculated blood osmolality were also lower in people with the highest levels of physical activity (Table 4).

### 4.2. Effects of Climate on Physical Activity, Water Intake/Fluid Loss and Hydration

We found an interaction effect between physical activity and climate. Physical activity (estimated by IPAQ) was reduced in the summer and this reduction was associated with increased maximal ambient temperatures (*r* = −0.277; *p* < 0.001; Table 3). In contrast, most of the available data show that physical activity increases from winter to spring or summer [35]. However, these studies were conducted in places with moderate summer temperatures like Ontario, Massachusetts, Glasgow, Netherlands, Central Japan or Aberdeen [35]. The high temperatures in Toledo and Athens in the summer may have been responsible for the currently reported reduction summer physical activity. This environmentally mediated reduction in physical activity in the summer, did not prevent the occurrence of higher non-renal water losses likely belonging to increased sweating (*r* = 0.147; *p* < 0.001). Maximal ambient temperature was associated with reduced urine output and increased urine osmolality (*r* = 0.218; *p* < 0.001; Table 3). Blood also responded to ambient temperature with increased concentration (Table 4). Our interpretation of these data is that environmental heat acts to reduce voluntary physical activity but does not prevent higher sweat losses that in the face of an insufficient increase in water intake, results in moderate dehydration. When data is analyzed using winter-summer category instead of using the continuous maximal daily temperature, urine osmolality (dehydration index) was not higher in the summer (Table 1). One factor that could explain the lack of effect of season in urine osmolality is that while in Spain and Greece maximal temperatures during summer likely induce sweating and dehydration, in Germany summer temperatures are lower and less dehydrating. Thus, the winter-summer classification may not be ideal when testing subjects in different latitudes.

Although in the short term, physical activity results in water loss (*i.e.*, exercise induce-sweating), it is less clear what is the result of different levels of physical activity on 24-h water intake and loss. In a thorough study, Westerterp and co-workers studied 42 women and 10 Dutch men (all lean and young) during 7 days. They were tested for water intake (weighted record of foodstuff and beverage), physical activity (difference between total and resting energy expenditure) and water loss (deuterium elimination method) in summer and winter [9]. The study revealed that in men, water loss was higher in subjects with a higher physical activity regardless of the season. However, in women water loss was higher in summer but was unrelated to physical activity [9]. In another study, water turnover was measured in rural and urban women in Kenya. The authors found that that BMI was the strongest predictor of water loss. However, the addition of physical activity (measured by accelerometry) to the prediction equation accounted for an additional 12% of the variance in water loss [10]. Our study in a much larger and heterogeneous subject sample confirms a positive association between physical activity and water loss (urine volume) but also a positive association with 24-h water intake (Table 3). Seemingly this higher water turnover results in improved hydration status with lower urine and blood osmolality in the subjects with higher physical activity levels.

### 4.3. Effects of Aging on Hydration 

Our older groups of participants (40–50 and 50–60 years’ old) seemed to be able to maintain a level of physical activity similar to the younger age groups (Table 1). This project was housed in three Universities and although it was meant to reach all population segment and types from the surrounding cities (Cologne, Athens and Toledo) the socioeconomic status of the inhabitants in these urban, university-cities is moderate to high. This population receive and can better implement the health advices to promote physical activity for healthy aging than in the rural areas. From this perspective, it is not surprising the reported maintenance of physical activity in the older segments of our sample. Furthermore, the age group of 20–30 years’ old tended to be the less physically active group (Table 1) likely due to high time-consuming nature of studying/starting in a new job. The similar physical activity in the older subjects did not prevent the decrease in the capacity for renal water reabsorption described in previous studies [21,22]. Reduction in the capacity to concentrate urine is supported in our result of higher urine output and lower urine osmolality with increases in age ([21]; Table 1).

### 4.4. Effects of Gender and Country of Residency on Hydration 

Men displayed higher 24-h water intake, NRWL (*i.e.*, sweating) and urine osmolality than the women of our sample. The larger average body size linked to higher energy expenditure/consumption could explain the highest sweat loss and fluid intake in men. However, there is no ready explanation for the gender effect on urine osmolality. The chief metabolite in urine is urea, which is derived from protein catabolism. On average, men have more muscle mass, protein turnover and thus may need to clear more urea in urine. We recently found that men with higher amount of muscle mass (rugby players) have higher urine osmolality than men with lower muscle mass (endurance runners) despite similar hydration status [36]. Alternatively, men may consume more protein than women influencing urine osmolality. Thus, the higher urine osmolality in men in comparison to women (Table 1) does not necessarily mean that the men in our sample were hypohydrated in comparison to the women but rather that urine osmolality is influenced by factors apart from hydration.

We also found significant country differences the most obvious being the lower maximal temperature in Germany in comparison to Spain and Greece (17 °C *vs.* 24–25 °C when averaging summer and winter data). Our German subjects were more physically active and there was a significant correlation between higher maximal ambient temperatures and reduced physical activity. Thus, our data suggests that the higher ambient temperatures in Spain and Greece may have inhibited physical activity in comparison to Germany. Besides environmental heat, differences in city architectural design to allow physical activity engagement (*i.e.*, parks, bike-lanes, pedestrian paths) and people’s knowledge of the impact of exercise in their health, could also contribute to the higher PA found in German subjects. German subjects also ingests more fluid per day and had a higher urine volume. These data corroborates our finding of a link between PA and water turnover even when data are analyzed by countries.

### 4.5. Limitations of the Study 

There are some limitations in our study that should be kept in mind. Water intake, urine volume and physical activity were all self-reported values. These variables are subjected to participant under or over-reporting. To avoid misreporting, subjects were instructed to record drinks and food ingested immediately after it happened but some underreporting in weekends was conceivable. Subjects were instructed to collect each void in a plastic jar, and record the urine weight before disposal. Likewise than with the food and drink diary, underreporting was possible. Completeness of 24-h urine collection could have been assessed by urine analysis of the recovery of p-aminobenzoic acid previously ingested [37]. However, this biochemical analysis was not available in our facilities. Subjects recorded physical activity at night by filling out a daily log and thus this measurement was subjected to individual’s recall ability. Other means to track physical activity ordered by precision are pedometers, accelerometers or double-labelled water, which were not available in our study. Lastly, our correlations (Table 3 and Table 4) only point to associations between variables and manipulative studies are due to establish cause-effect among these factors. Furthermore, we ought to recognize that although significant, many correlations among variables were small (*r* < 0.3) and the conclusions based on those are tentative.

## 5. Conclusions

Our data compiling seven consecutive testing days on 573 men and women aged 20–60 years’ old suggests an association between elevated ambient temperatures and lowering physical activity with a larger effect in the southern European countries (Spain and Greece) in comparison to northern Europe (Germany). The reduction in physical activity in summer did not prevent higher non-renal water loss (*i.e.*, mostly sweating) and hypohydration despite increased water intake. When summer and winter data were compiled, better hydration (lower urine and plasma osmolality) was associated with elevated levels of physical activity (IPAQ). This suggests that the exercise training adaptations to expand body water and improve hydration status may also occur in the general population, mostly in those with high levels of physical activity. Finally, our results confirm previous reports in that aging reduce the capacity to concentrate urine.

## Figures and Tables

**Table 1:** Effects of gender, season, site, age group and hydration on physical activity and 24-h water intake/fluid loss.

	IPAQ (MET-min/Week)	Max Air Temp (°C)	Water Intake (L/Day)	Urine Volume (L/Day)	Non-Renal Water Loss (L/Day)	Urine Osmol (mOsmol/kg)
Female	2356 ± 1774	22 ± 9	2.57 ± 0.89	1.66 ± 0.74	0.89 ± 0.56	585 ± 228
Male	2141 ± 1475	21 ± 9	2.93 ± 1.00 *	1.64 ± 0.66	1.31 ± 0.94 *	674 ± 203 *
Winter	2571 ± 2018	14 ± 6	2.64 ± 0.98	1.67 ± 0.66	0.99 ± 0.87	615 ± 208
Summer	1942 ± 1087 *	29 ± 4 *	2.85 ± 1.03 *	1.62 ± 0.73	1.21 ± 0.71 *	646 ± 230
Germany	2945 ± 2053	17 ± 8	3.29 ± 0.98	2.13 ± 0.75	1.16 ± 0.62	492 ± 170
Spain	2088 ± 988 *	24 ± 8 *	2.56 ± 1.01 *	1.40 ± 0.49 *	1.14 ± 1.00	754 ± 179 *
Greece	1422 ± 1200 *^,†^	25 ± 8 *	2.34 ± 0.77 *	1.35 ± 0.49 *	1.02 ± 0.72	658 ± 224 *^,†^
20–30 years old	2103 ± 1196	21 ± 9	2.68 ± 1.12	1.49 ± 0.72	1.18 ± 0.81	686 ± 220
30–40 years old	2327 ± 1782	23 ± 8	2.75 ± 0.91	1.69 ± 0.71	1.07 ± 0.65	613 ± 226 *
40–50 years old	2176 ± 1810	22 ± 9	2.86 ± 0.96	1.70 ± 0.69 *	1.18 ± 0.75	621 ± 224 *
50–60 years old	2420 ± 1722	22 ± 8	2.71 ± 1.05	1.74 ± 0.63 *	0.96 ± 0.95	590 ± 199 *
Urine >500 (mOsm/kg)	2086 ± 1408	23 ± 9	2.53 ± 0.94	1.35 ± 0.46	1.19 ± 0.85	750 ± 155
Urine <500 (mOsm/kg)	2563 ± 1984 *	20 ± 8 *	3.20 ± 1.01 *	2.25 ± 0.72 *	0.94 ± 0.65 *	377 ± 79 *
(−) FWR	1983 ± 1137	25 ± 8	2.38 ± 1.02	1.10 ± 0.31	1.24 ± 0.94	891 ± 104
(+) FWR	2362 ± 1787 *	21 ± 9 *	2.92 ± 0.98 *	1.87 ± 0.69 *	1.04 ± 0.73 *	520 ± 153 *

Data represents average of 7 days data collection ± SD; *, different from the first value listed in that cell; ^†^, different from the value above in cells that contain more than two values (*i.e.*, country and age group); all *p* < 0.05; FWR, stands for Free Water Reserve.

**Table 2:** Sample size in categorical variables and percentage in each category.

	Physical Activity (% Subjects)	Maximal Air Temp °C (% Subjects)
Gender		
Female	249 (50%)	282 (50%)
Male	252 (50%)	284 (50%)
Total	501	566
Season		
Winter	240 (47%)	272 (47%)
Summer	266 (53%)	301 (53%)
Total	506	573
Country		
Germany	188 (37%)	201 (36%)
Spain	192 (38%)	193 (34%)
Greece	126 (25%)	170 (30%)
Total	506	564
Age groups		
20–30 years’ old	140 (28%)	155 (27%)
30–40 years’ old	123 (25%)	139 (25%)
40–50 years’ old	125 (25%)	138 (24%)
50–60 years’ old	113 (22%)	132 (24%)
Total	501	564
Urine Osmol		
>500 mOsmol/kg	343 (68%)	384 (67%)
<500 mOsmol/kg	162 (32%)	187 (33%)
Total	505	571

Average sample size of 536 individuals.

**Table 3:** Correlation (*r* Pearson) among physical activity, maximal air temperature, 24-h water intake/fluid loss and urine osmolality.

	IPAQ (MET-min/Week)	Max Air Temp (°C)	Water Intake (L/Day)	Urine Volume (L/day)	Non-Renal Water Loss (L/Day)	Urine Osmolality (mOsmol/kg)
IPAQ (MET-min/week)	-------	−0.283	0.145	0.158	NS	−0.151
		*p* < 0.001	*p* < 0.001	*p* < 0.001		*p* = 0.001
Max Air Temp (°C)		-------	NS	−0.222	0.147	0.218
				*p* < 0.001	*p* < 0.001	*p* < 0.001
Water Intake (L/day)			-------	0.623	0.716	−0.349
				*p* < 0.001	*p* < 0.001	*p* < 0.001
Urine Volume (L/day)				-------	−0.095	−0.736
					*p* = 0.024	*p* < 0.001
Non-Renal Water Loss (L/day)					-------	0.208
						*p* < 0.001
Urine Osmolality (mOsmol/kg)						-------

*r* correlation values are listed in bold and *p* values below; NS, stands for non-significant.

**Table 4:** Correlation (*r* Pearson) between blood variables and physical activity, air temperature and 24-h water intake/ fluid loss.

	IPAQ (MET-min/Week)	Max Air Temp (°C)	Water Intake (L/Day)	Urine Volume (L/Day)	Non-Renal Loss (L/Day)
Serum [Na^+^] (mEq/L)	−0.145	0.189	−0.118	−0.115	NS
	*p* < 0.003	*p* < 0.001	*p* = 0.006	*p* < 0.005	
Serum [K^+^] (mEq/L)	−0.098	0.154	NS	NS	NS
	*p* = 0.040	*p* < 0.001			
Blood Glucose (mmol/L)	−0.125	0.194	NS	−0.115	NS
	*p* = 0.023	*p* < 0.001		*p* = 0.007	
Serum Osmolality calculated (mOsmol/kg)	−0.137	0.163	−0.123	−0.112	NS
	*p* = 0.004	*p* < 0.001	*p* = 0.008	*p* = 0.015	
Blood hemoglobin (g/L)	NS	NS	NS	NS	0.174
					*p* < 0.001

*r* correlation values are listed in bold and *p* values below, NS, stands for non-significant.
